# Regulatory effects of Hemin on prevention and rescue of salt stress in rapeseed (*Brassica napus L.*) seedlings

**DOI:** 10.1186/s12870-023-04595-z

**Published:** 2023-11-14

**Authors:** Hui-Min Zhao, Dian-Feng Zheng, Nai-Jie Feng, Guang-Sheng Zhou, Aaqil Khan, Xu-Tong Lu, Peng Deng, Hang Zhou, You-Wei Du

**Affiliations:** 1 College of Coastal Agriculture Sciences, Guangdong Ocean University, Zhanjiang, 524088 China; 2 South China Center of National Saline-tolerant Rice Technology Innovation Center, Zhanjiang, 524088 China; 3 Shenzhen Research Institute of Guangdong Ocean University, Shenzhen, 518108 China; 4 College of Plant Science & Technology of Hua Zhong Agricultural University, Wuhan, 430070 China

**Keywords:** Antioxidant system, Hemin, Photosynthesis, Salinity stress, Rapeseed

## Abstract

**Background:**

Salt stress severely restricts rapeseed growth and productivity. Hemin can effectively alleviate salt stress in plants. However, the regulatory effect of Hemin on rapeseed in salt stress is unclear. Here, we analyzed the response and remediation mechanism of Hemin application to rapeseed before and after 0.6% (m salt: m soil) NaCl stress. Experiment using two *Brassica napus* (AACC, 2n = 38) rapeseed varieties Huayouza 158R (moderately salt-tolerant) and Huayouza 62 (strongly salt-tolerant). To explore the best optional ways to improve salt stress resistance in rapeseed.

**Results:**

Our findings revealed that exogenous application of Hemin enhanced morph-physiological traits of rapeseed and significantly attenuate the inhibition of NaCl stress. Compared to Hemin (SH) treatment, Hemin (HS) significantly improved seedlings root length, seedlings height, stem diameter and accumulated more dry matter biomass under NaCl stress. Moreover, Hemin (HS) significantly improved photosynthetic efficiency, activities of antioxidant enzymes such as superoxide dismutase (SOD), peroxidase (POD), ascorbate peroxidase (APX), and decreased electrolyte leakage (EL) and malondialdehyde (MDA) content, thus resulting in the alleviation of oxidative membrane damage. Hemin (HS) showed better performance than Hemin (SH) under NaCl stress.

**Conclusion:**

Hemin could effectively mitigate the adverse impacts of salt stress by regulating the morph-physiological, photosynthetic and antioxidants traits of rapeseed. This study may provide a basis for Hemin to regulate cultivated rapeseed salt tolerance and explore a better way to alleviate salt stress.

## Background

Increased urbanization and seawater intrusion decreased arable land in coastal areas which has detrimental impacts on agricultural production [1]. Worldwide salinity stress is the most extensive and influential abiotic stress that has adverse impacts on crop growth and productivity. Salinity accounts for about 7% of the world’s total land and about 20% of irrigated land [2]. Salinity has deleterious impacts on plant physiology and biochemistry, thus creating risks to agricultural productivity and food security.

High salt concentration in the soil leads to three kinds of interacting stress. These are ionic stress caused by ionic toxicity (especially Na^+)^, oxidative stress mainly caused by reactive oxygen species (ROS) accumulation, and osmotic stress caused by water deficit [3, 4]. Surplus ions induce stomatal closure to reduce carbon dioxide concentration, and decrease water loss through transpiration, which inhibits net photosynthetic rate [5]. Plant cells and tissues typically possess well organized enzymatic ROS scavenging systems, including superoxide dismutase (SOD), peroxidase (POD), catalase (CAT) and ascorbate peroxidase (APX), as well as non-enzymatic antioxidants, glutathione (GSH) and ascorbate (ASA), liable to quench ROS and alleviate the damages to photosynthetic membrane caused by salt stress [6, 7].

Rapeseed (*Brassica napus.*) is one of the most vital oil-seeds crop globally, grown as meal protein for humans and animal consumption [8]. This crop is very sensitive to salt stress throughout the plant growth and development. Salinity stress impacts rapeseed productivity, by impairing osmotic stress and ionic imbalance, which severely effects water uptake and net photosynthetic rate [9]. Direct seeding of rapeseed in saline soils can inhibit seed germination, growth and developmental cycle or even cause seedling death [10]. Therefore, rapeseed is usually nursed and transplanted to the field to ensure the consistency of harvest and yield [11]. To avoid these seedling death scenarios, the application of plant growth regulators could be effective in eliminate the adverse effects of abiotic stresses [12].

Hemin is a novel plant growth regulator and is naturally derived chloride of heme [13]. Primarily it can be used as an effective promoter of heme oxygenase (HO-1), broken down by HO-1 to carbon monoxide CO, biliverdin (BV) and ferrous ions Fe^2+^ [14, 15]. Previously Hemin was used in animal research but currently it is using in plants as a powerful tool that is vital in protecting plants from various abiotic stresses [16]. Literature showed that exogenous Hemin enhanced photosynthesis and reduce inhibition caused by salt stress by activating the antioxidant system such as SOD, POD and CAT [17, 18]. Hemin alleviated damages in wheat seedlings caused by high temperature [19], and enhanced the tolerance of *Arabidopsis thaliana* under salt stress by regulating ROS homeostasis [20, 21]. Previous studies focused Hemin alleviating heavy metal stress on *Brassica* [17, 22].However, its definite enactment and the inevitable mechanisms in alleviating salt damages and increasing salt tolerance in rapeseed needs to be clarified. And the studies comparing the effects of spraying Hemin before and after salt stress on the alleviation of salt stress in rapeseed seedlings are minimally. We hypothesis that Hemin would regulate differently during different periods of salt stress.

Two *Brassica napus* (*B. napus*; AACC, 2n = 38) were selected for this study: *B. napus* Huayouza 62 and *B. napus* Huayouza 158R. Foliar sprayed with Hemin before and 24 h after NaCl treatment, respectively. Through assess the potential role of Hemin in mediating the antioxidant system, osmoregulation, photosynthesis-related attributes, along with growth and biomass accumulation in rapeseed under salt stress. To elucidate the mechanisms by which Hemin improves salt tolerance in rapeseed seedlings under different salt stress stages, and to explore the optimal salt tolerance effect. The aim is to Provide new ideas for studying the effect of Hemin on salt tolerance in rapeseed and provide evidence for the practical application of Hemin in increasing salt tolerance of cultivated rapeseed in saline areas.

## Results

### Effect of foliar spraying of Hemin on growth parameters of rapeseed seedlings before and after NaCl stress

**Fig. 1:**
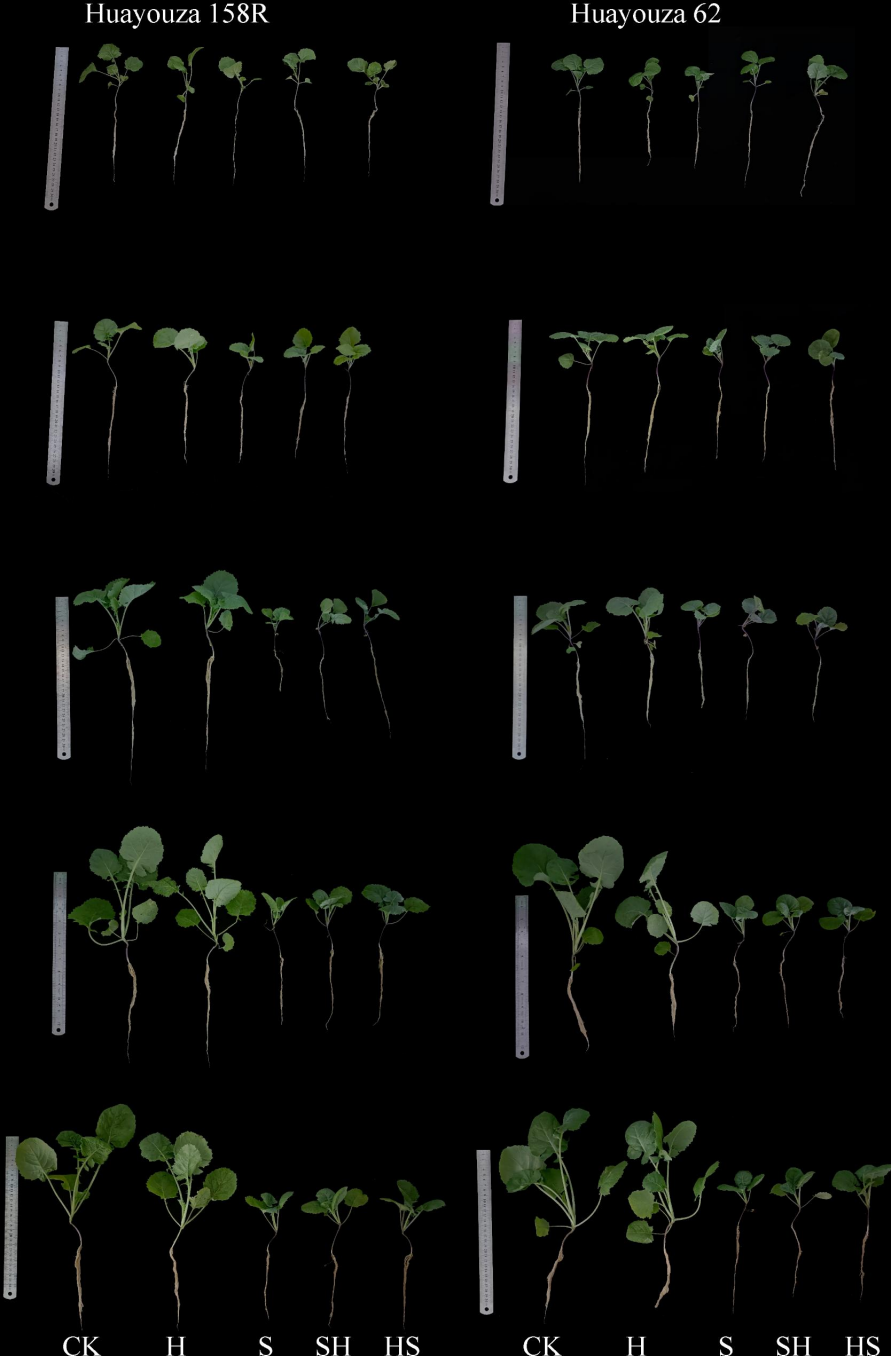
Effect of Hemin prophylaxis and treatment on plant morphology of rapeseed under NaCl stress. CK: Control; H: Hemin; S: NaCl treatment; SH: NaCl + Hemin; HS: Hemin + NaCl

The morphological traits such as root length, seedling height, stem diameter and leaf area were significantly reduced by NaCl stress (Table 1). As the NaCl treatment (S) was applied to plants, at the 1st-13th days, there were corresponding percent decreases in seedlings height of 16.8%, 26.3%, 38.8%, 52.5%, and 52.7% in Huayouza 158R, and 18.5%, 28.6%, 31%, 48.7% and 56% in Huayouza 62, respectively as compared to CK. Regardless of the treatment method, the application of exogenous Hemin (SH and HS) alleviated the inhibition of NaCl stress on the growth of rapeseed seedlings (Fig. 1). However, the current analysis showed that foliar application of exogenous Hemin (SH and HS) substantially increased root length, seedling height, stem diameter, and leaf area of rapeseed plants. At the 7th day, the application of Hemin (SH) increased percentage was 15.9% and 12.6% in root length, 4.8% and 10.5% in stem diameter, 23.1% and 12.6% in leaf area, respectively for Huayouza 158R and Huayouza 62. While the pretreatment of Hemin (HS) increased percentage was 27.8% and 21.5% in root length, 14.3% and 15.8% in stem diameter, 56.3% and 41.5% respectively for Huayouza 158R and Huayouza 62. The alleviating inhibitory effect of Hemin (HS) treatment was significant at day 7th. It means that in contrast to Hemin (SH), the treatment Hemin (HS) showed better performance in enhancing plant morphological traits under NaCl stress.

**Table 1:** Effect of Hemin prophylaxis and treatment on growth parameters of rapeseed under NaCl stress

Huayouza 158R	Index	Treatments	1	4	7	10	13
	Plant height	Control	13.7 ± 0.1a	16.0 ± 0.1a	19.6 ± 0.03a	25.5 ± 0.4a	25.8 ± 0.1a
		H	13.0 ± 0.2b	15.3 ± 0.03b	18.3 ± 0.3b	24.4 ± 0.3b	25.7 ± 0.5a
		S	11.4 ± 0.1c	11.8 ± 0.2d	12.0 ± 0.3d	12.1 ± 0.1d	12.2 ± 0.1d
		SH	12.0 ± 0.2c	12.8 ± 0.2c	13.2 ± 0.1c	13.5 ± 0.1c	14.5 ± 0.03b
		HS	11.5 ± 0.3c	12.0 ± 0.03d	12.5 ± 0.1d	13.0 ± 0.2 cd	13.2 ± 0.3c
	Root length	Control	13.0 ± 0.2ab	14.9 ± 0.2b	18.4 ± 0.6b	19.3 ± 0.7b	21.3 ± 1.8a
		H	13.5 ± 1.0a	16.2 ± 0.1a	20.0 ± 0.5a	21.6 ± 0.2a	22.0 ± 0.4a
		S	11.2 ± 0.2c	12.2 ± 0.4c	12.6 ± 0.2e	14.4 ± 0.1d	14.7 ± 0.3b
		SH	12.6 ± 0.5ab	13.3 ± 0.6c	14.6 ± 0.2d	15.3 ± 0.4 cd	16.5 ± 0.1b
		HS	13.0 ± 0.6ab	14.7 ± 0.2b	16.1 ± 0.6c	16.5 ± 0.3c	16.9 ± 0.49b
	Steam diameter	Control	2.1 ± 0a	2.4 ± 0.1b	3.0 ± 0.03b	3.1 ± 0.03b	3.3 ± 0.1a
		H	2.1 ± 0a	2.8 ± 0.03a	3.2 ± 0.03a	3.3 ± 0.03a	3.3 ± 0.1a
		S	1.8 ± 0c	2.0 ± 0.1c	2.1 ± 0.03d	2.2 ± 0.1d	2.2 ± 0.1c
		SH	2.0 ± 0.030b	2.2 ± 0.1bc	2.2 ± 0.03d	2.3 ± 0.03d	2.4 ± 0.03bc
		HS	2.00 ± 0.030a	2.3 ± 0.1b	2.4 ± 0.03c	2.4 ± 0.03c	2.6 ± 0.1b
	Leaf area	Control	2662.7 ± 63.2a	4363.7 ± 58.2a	15071.2 ± 161.0a	16517.1 ± 654.9a	17720.0 ± 418.4a
		H	2386.1 ± 134.1ab	4812.6 ± 183.0a	12075.3 ± 148.2b	16047.0 ± 295.1a	17705.5 ± 1185.1a
		S	1897.1 ± 129.04c	2503.5 ± 62.0c	2567.3 ± 45.2e	3120.1 ± 85.9c	3581.8 ± 141.0b
		SH	2195.1 ± 98.1bc	2571.6 ± 178.9c	3159.6 ± 119.5d	3762.7 ± 67.8bc	5219.7 ± 283.7b
		HS	2286.8 ± 101.5b	3533.6 ± 415.9b	4012.9 ± 76.3c	4975.7 ± 504.2b	5434.9 ± 358.0b
Huayouza 62							
	Plant height	Control	12.4 ± 0.2a	15.4 ± 0.0a	17.1 ± 0.2a	23.4 ± 0.2a	28.4 ± 0.2a
		H	12.0 ± 0.3a	14.8 ± 0.1a	16.2 ± 0.1b	23.3 ± 0.1a	24.9 ± 0.2b
		S	10.1 ± 0.2b	11 ± 0.5c	11.8 ± 0.2d	12 ± 0.1c	12.5 ± 0.1d
		SH	10.8 ± 0.4b	12.5 ± 0.2b	12.8 ± 0.2c	13.3 ± 0.1b	13.6 ± 0.1c
		HS	10.5 ± 0.0b	12.3 ± 0.1b	12.5 ± 0.5 cd	13.1 ± 0.1b	13.3 ± 0.3c
	Root length	Control	15.2 ± 0.1a	15.7 ± 0.3b	18.5 ± 0.5a	18.8 ± 0.2a	20.1 ± 1.1a
		H	14.9 ± 0.7a	16.8 ± 0.2a	18.5 ± 0.4a	18.9 ± 0.3a	21.0 ± 1.3a
		S	12.1 ± 0.3c	12.9 ± 0.1d	13.5 ± 0.6c	14.2 ± 0.4d	14.9 ± 0.1b
		SH	13.5 ± 0.2b	14.2 ± 0.1c	15.2 ± 0.1b	15.8 ± 0.4c	16.1 ± 0.7b
		HS	14.3 ± 0.1ab	14.6 ± 0.3c	16.4 ± 0.1b	16.9 ± 0.4b	17.5 ± 0.3b
	Steam diameter	Control	2.1 ± 0.03a	2.4 ± 0.03b	2.5 ± 0.03a	2.6 ± 0.03b	3.1 ± 0.03b
		H	2.2 ± 0.03a	2.6 ± 0.1a	2.7 ± 0a	2.8 ± 0.1a	3.2 ± 0a
		S	1.8 ± 0.1c	1.9 ± 0.1c	1.9 ± 0.1c	2 ± 0.1d	2.1 ± 0.03d
		SH	1.9 ± 0bc	2.1 ± 0.03c	2.1 ± 0.1bc	2.1 ± 0.03 cd	2.3 ± 0.1c
		HS	2.0 ± 0.1b	2.1 ± 0.1c	2.2 ± 0.1b	2.3 ± 0.03c	2.4 ± 0.03c
	Leaf area	Control	3783.3 ± 267.0a	6179.9 ± 103.8a	9367.6 ± 63.7a	15904.3 ± 143.7a	20673.4 ± 249.0a
		H	3234.9 ± 151.9b	5987.7 ± 85.4a	8994.2 ± 57.5a	13758.8 ± 266.4b	19273.8 ± 464.0b
		S	2162.4 ± 58.7c	2651.4 ± 57.1d	2882.9 ± 193.4c	4051.4 ± 157.2d	5400.4 ± 149.8d
		SH	2319.9 ± 29.1c	2908.0 ± 101.8c	3247.2 ± 147.9c	4489.1 ± 42.6 cd	5595.1 ± 133.6 cd
		HS	2933.7 ± 17.4b	3218.8 ± 24.9b	4079.9 ± 238.1b	4610.6 ± 71.6c	6330.5 ± 52.5c

CK: Control; H: Hemin; S: NaCl treatment; SH: NaCl + Hemin; HS: Hemin + NaCl. Mean ± SE of three replicates. Different letters indicate significant differences (p < 0.05)

### Effects of foliar spraying Hemin before and after NaCl stress on biomass of Rape seedlings

**Fig. 2:**
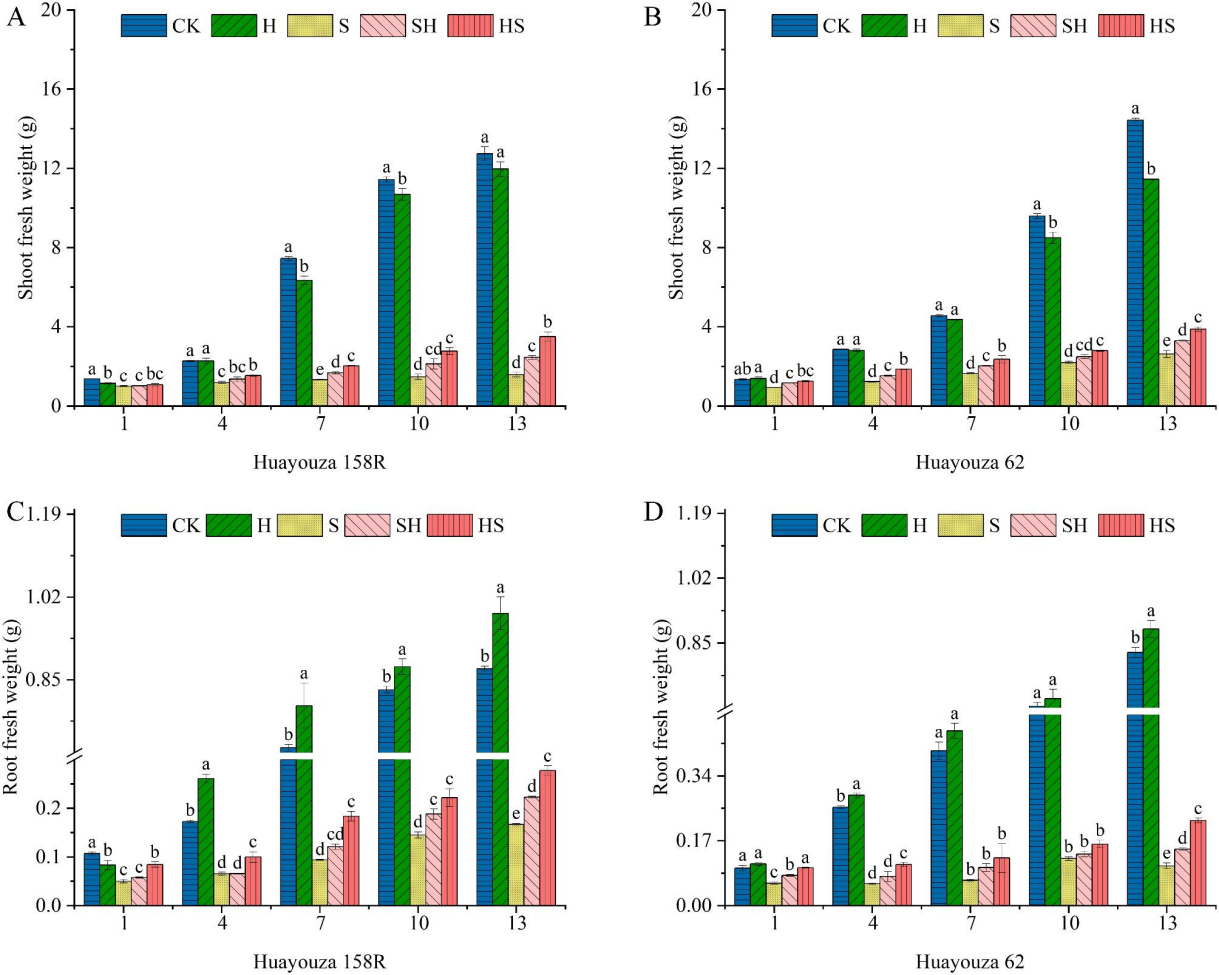
Effect of Hemin prophylaxis and treatment on fresh weight of rapeseed above (**A**, **B**) and below ground (**C**, **D**) under NaCl stress. Mean ± SE of three replicates. CK: Control; H: Hemin; S: salt treatment; SH: NaCl + Hemin; HS: Hemin + NaCl. Different letters indicate significant differences (*p* < 0.05)

**Fig. 3:**
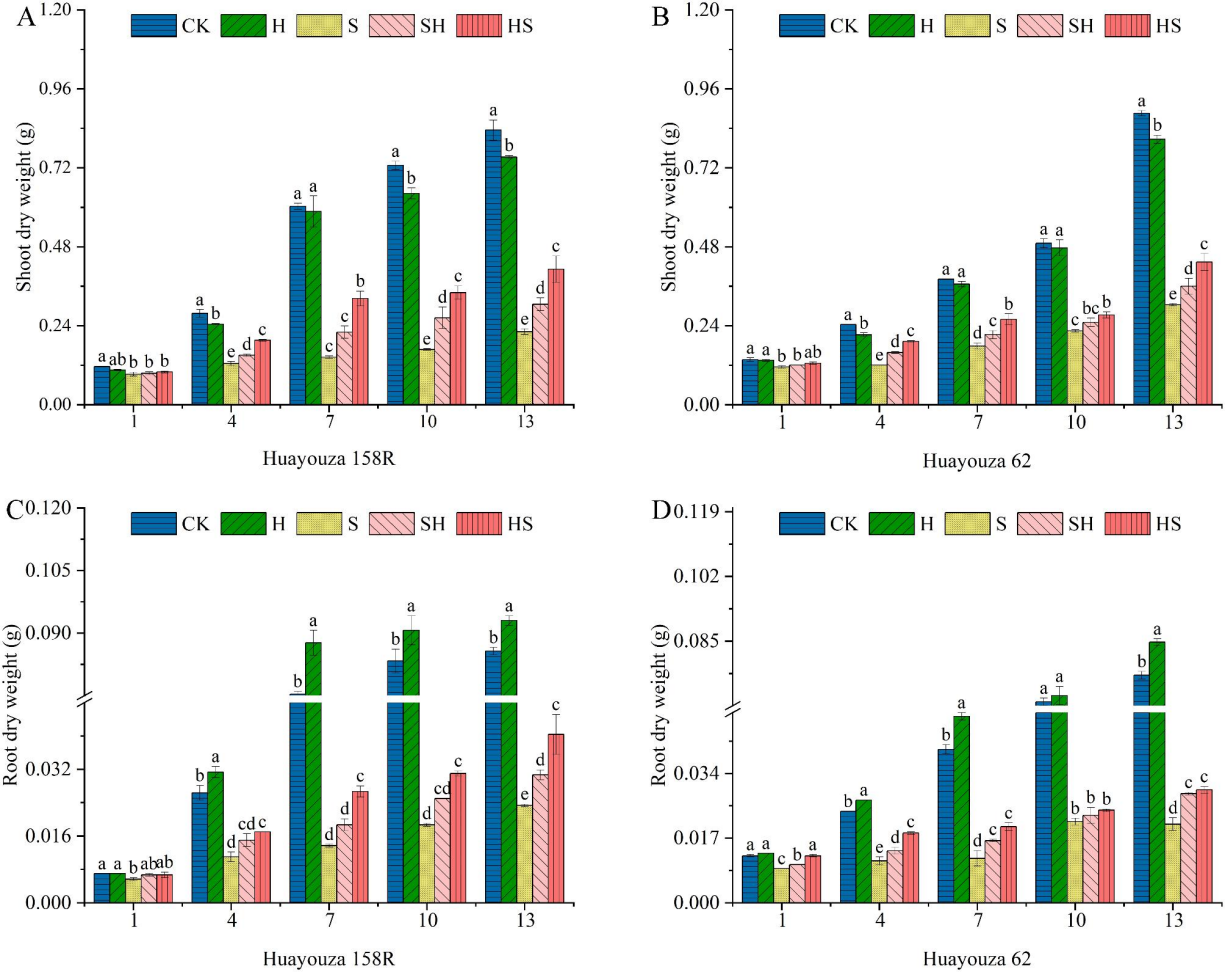
Effect of Hemin prophylaxis and treatment on the dry weight of rapeseed upper (**A**, **B**) and lower ground (**C**, **D**) of rapeseed under NaCl stress. CK: Control; H: Hemin; S: NaCl treatment; SH: NaCl + Hemin; HS: Hemin + NaCl. Mean ± SE of three replicates. Different letters indicate significant differences (*p* < 0.05)

With the increased of treatment time, NaCl treatment significantly inhibited the biomass accumulation of rapeseed seedlings (Figs. 2 and 3). The most significantly decreased percentage in shoot and root dry weight was 77.0% and 77.8% in Huayouza 158R on day 10, and 65.71% and 72.8% (Fig. 3) in Huayouza 62, on day 13, as compared to CK. The application of exogenous Hemin increased the fresh and dry weights of seedlings shoot and root compared with NaCl treatment alone, which led to the alleviation of the inhibition of seedling growth by NaCl, and the foliar spraying of Hemin was more effective before being subjected to NaCl stress (Figs. 2 and 3). While the shoot and root dry matter weight increased percentage were 123.6% and 95.1% in HS treatment, and 52.9% and 36.6% in SH treatment, for Huayouza 158R. In Huayouza 62, the shoot and root dry weights were increased by 45.3% and 71.4% in HS treatment, and 19.0%, and 40%, in SH treatment. The above ground and root fresh weights had the similar trend. On the 7th day, at the SH treatment, there was percentage increases of 26.1% and 23.0% in shoot fresh weight, and 28.7% and 51.0% in root fresh weight, for Huayouza 158R and Huayouza 62. While HS treatment increased percentage was 53.0% and 42.9% in shoot fresh weight, and 95.4% and 88.9% in root fresh weight, for Huayouza 1588R and Huayouza 62.

### Effects of foliar spraying Hemin before and after NaCl stress on membrane lipid peroxidation of rape seedlings

**Fig. 4:**
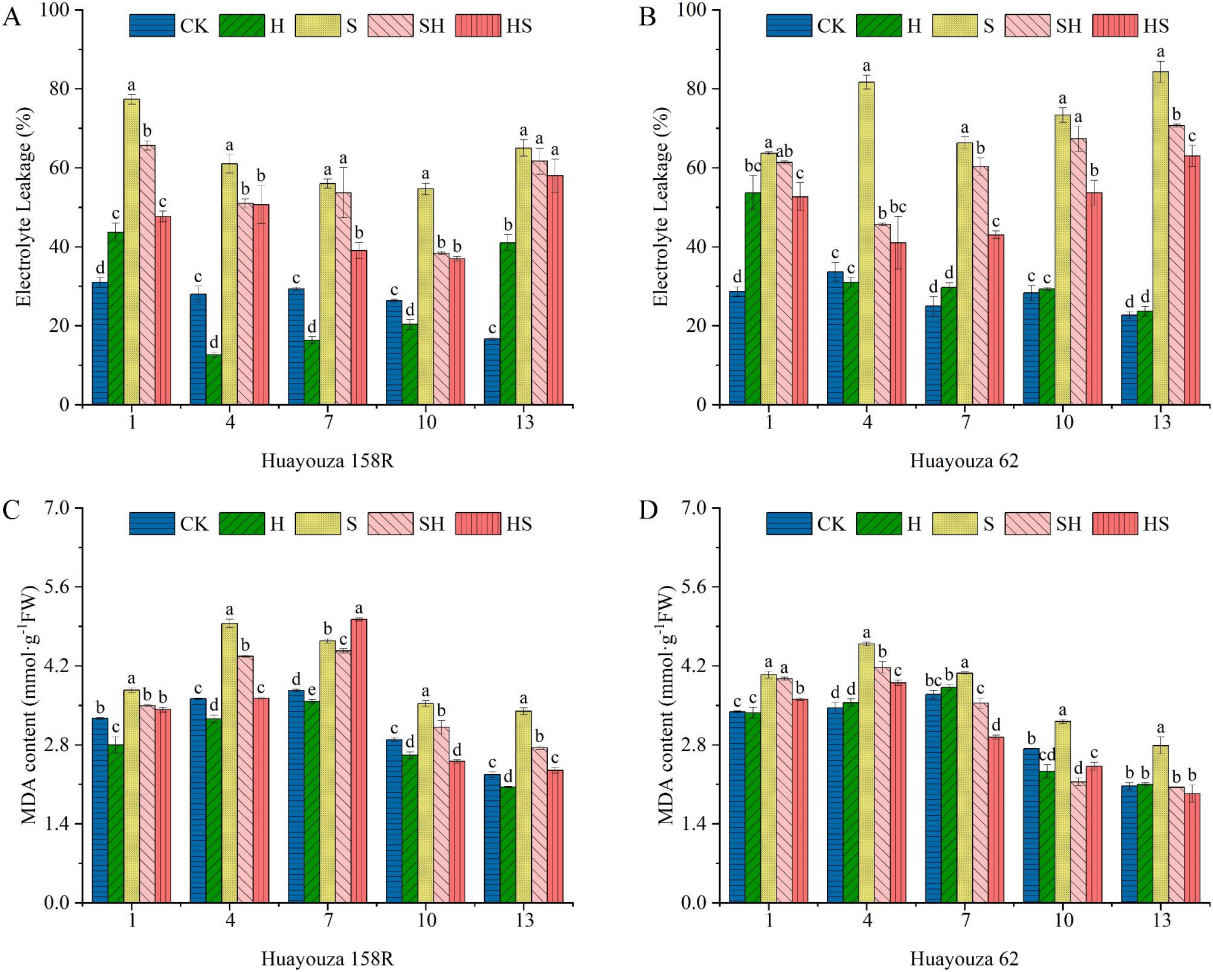
Effect of Hemin prophylaxis and treatment on electrolyte leakage rate (EL) (**A**, **B**) and malondialdehyde (MDA) (**C**, **D**) in rapeseed leaves under NaCl stress. CK: Control; H: Hemin; S: NaCl treatment; SH: NaCl + Hemin; HS: Hemin + NaCl. Mean ± SE of three replicates. Different letters indicate significant differences (*p* < 0.05)

As the NaCl treatment was applied to plants, at the 1st-7th days, the average percentage were increased of 151.2% and 29.4% in EL and 172.2%, 23.0% in MDA for Huayouza 158R and Huayouza 62. The HS treatment, at 1st-13th days, there were corresponding percent significantly decreases in EL of 38.4%, 16.9%, 30.4%, 32.3% and 10.8% in Huayouza 158R, and 17.3%, 49.8%, 35.2%, 26.8% and 25.3% in Huayouza 62 (Fig. 4C, D), respectively as compared with NaCl (S) treatment. The SH treatment, at 1st-13th days, there were corresponding percent significantly decreases in EL of 15.1%, 16.4%, 4.2%, 29.9% and 5.1% in Huayouza 158R, and 3.7%, 44.1%, 9.0%, 8.2% and 16.2% in Huayouza 62. On the 13th day, the MDA content most significantly decreased by 19.1% (SH) and 30.9% (HS) in Huayouza 158, and 26.6% (SH), and 30.6% (HS) in Huayouza 62, compared with NaCl (S) treatment. It means that the content of EL and MDA under stress was significantly reduced by Hemin treatment, and the decrease in Hemin pretreatment (HS) was significantly higher than that of rescue (SH) (except for MDA on the 7th day of Huayouza 62).

### Effect of foliar spraying of Hemin on photosynthetic parameters of rapeseed seedlings before and after NaCl stress

**Fig. 5:**
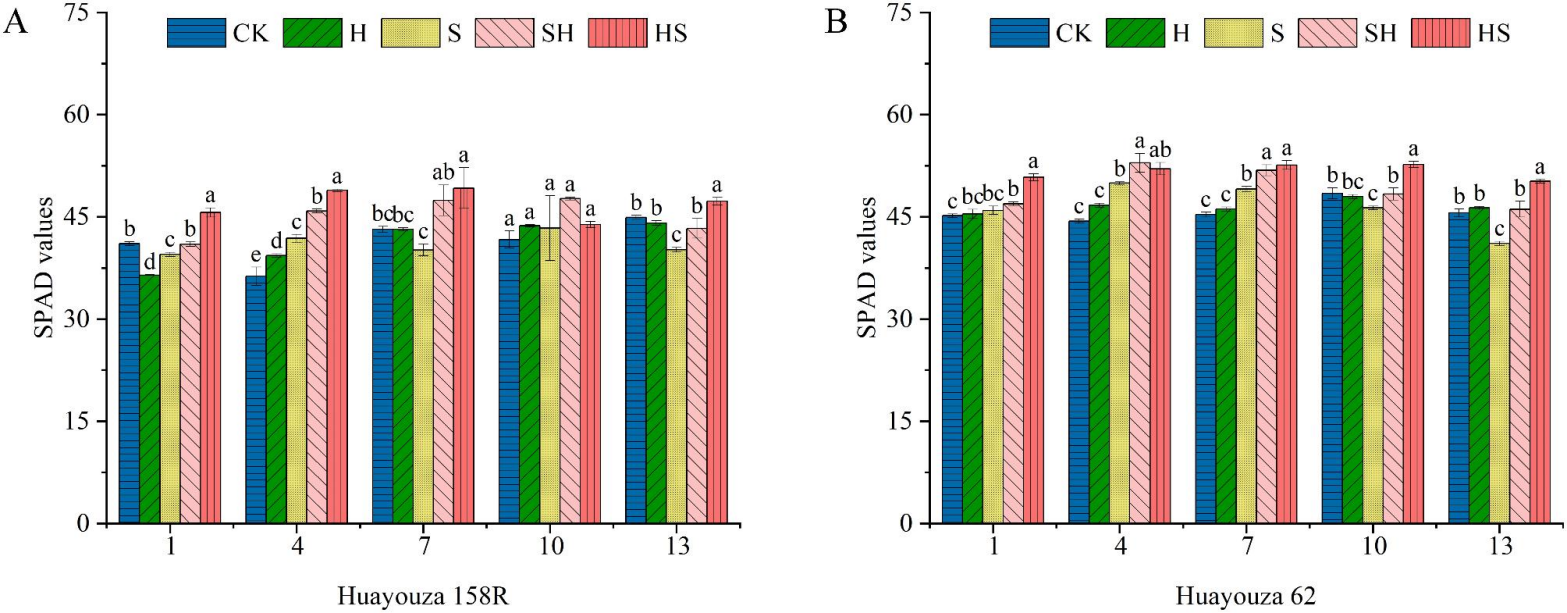
Effect of Hemin prophylaxis and treatment on rapeseed’s relative chlorophyll content (SPAD) (**A**, **B**) under NaCl stress. CK: Control; H: Hemin; S: NaCl treatment; SH: NaCl + Hemin; HS: Hemin + NaCl. Mean ± SE of three replicates. Different letters indicate significant differences (*p* < 0.05)

Under NaCl stress, the SPAD values decreased by 7.3% at the 7th day, and 10.6% at the 13th day of Huayouza 158R, compared with CK (Fig. 5). The SPAD values reduced by 4.4% at the 10th and 9.8% at the 13th day, respectively of Huayouza 62. Foliar application of Hemin increased by 3.8%, 9.6%, 18.2%, 10.0% and 7.8% in SH and 15.6%, 16.8%, 22.6%, 1.2% and 17.8% in HS of Huayouza 158R, at days 1st-13th, compared to S treatment. The SPAD values increased by 2.1%, 6.0%, 5.7%, 4.4% and12.2% in SH, and 10.6%, 4.2%, 7.2%, 13.7% and 22.2% in HS, respectively of Huayouza 62. In summary, HS treatment was more effective than SH treatment in alleviating salt stress.

**Fig. 6:**
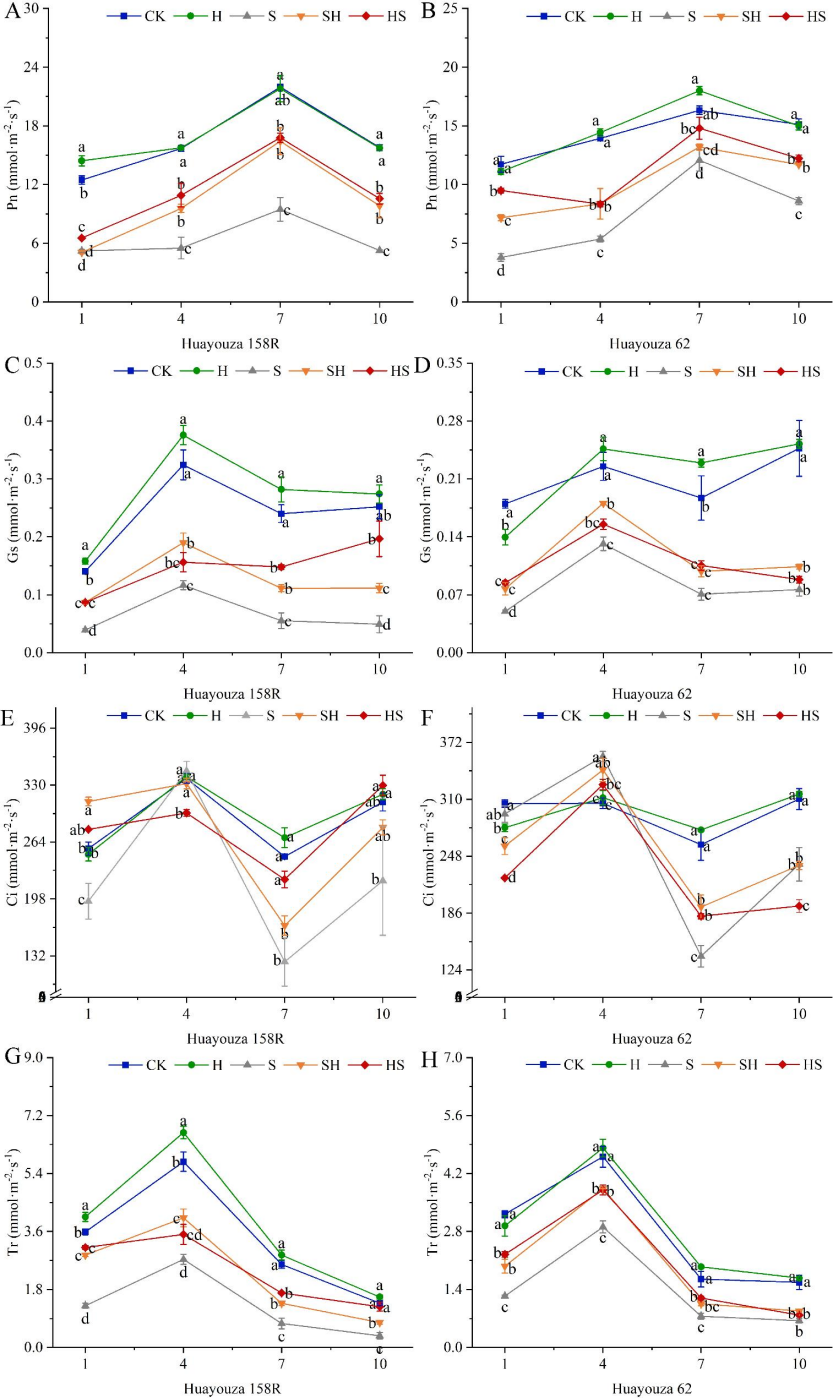
Effect of Hemin prophylaxis and treatment on net photosynthetic rate (Pn) (**A**, **B**), stomatal conductance (Gs) (**C**, **D**), inter-cellular CO_2_ concentration (Ci) (**E**, **F**), and transpiration rate (Tr) (**G**, **H**) in rapeseed under NaCl stress. CK: Control; H: Hemin; S: NaCl treatment; SH: NaCl + Hemin; HS: Hemin + NaCl. Mean ± SE of three replicates. Different letters indicate significant differences (*p* < 0.05)

Photosynthetic indicators such as net photosynthetic rate (Pn), stomatal conductance (Gs), and transpiration rate (Tr) were significantly decreased by NaCl stress (Fig. 6). From the 1st day to the 10th day, decreased Pn by 58.2%, 65.0%, 56.9%, 66.7% and 67.7%, 61.5%, 26.1%, 43.2%, Gs by 72.2%, 64.0%, 77.0%,80.4% and 72.2%, 41.6%, 62.2%, 69.1%, Tr by 63.9%, 52.5%, 71.2%, 73.6% and 61.5, 36.8%, 54.3%, 58.9% in Huayouza 158R and Huayouza 62 (Fig. 6). Except for the increase of Ci content under NaCl stress at the 4th day, it decreased significantly at the 1st, 7th and 10th days. The average percentage of Ci was reduced by 34.2% and 24.4% in Huayouza 158R and Huaouza 62, respectively (Fig. 6G, H). Compared with NaCl treatment, Hemin (HS) pretreatment increased percentage was 25.3%, 98.2%, 77.5%, 100.6% and 150.4%, 55.3%, 22.7%, 42.3% in Pn, 123.1%, 34%, 1667.5%, 298.7% and 169.3%, 18%, 49.1%, 15.7% in Gs, 139.3%, 28.3%, 127.4%, 248.6% and 81.2%, 30.7%, 58.4%, 20.6% in Tr, respectively for Huayouza 158R and Huayouza 62. And Hemin (HS) pretreatment was generally higher than that of spraying Hemin (SH) during the stress period, but it did not reach a significant level. Foliar spraying of Hemin (SH and HS) significantly increased Ci compared with NaCl treatment. On the contrary, Hemin treatment was higher than NaCl (S) treatment only on the 7th day in Huayouza 62. Hemin (SH and HS) treatments were significantly higher than NaCl alone, alleviating the inhibition of photosynthesis and having a positive effect on seedling growth.

### Effects of foliar spraying Hemin on antioxidant enzyme activity of rape seedlings before and after NaCl stress

**Fig. 7:**
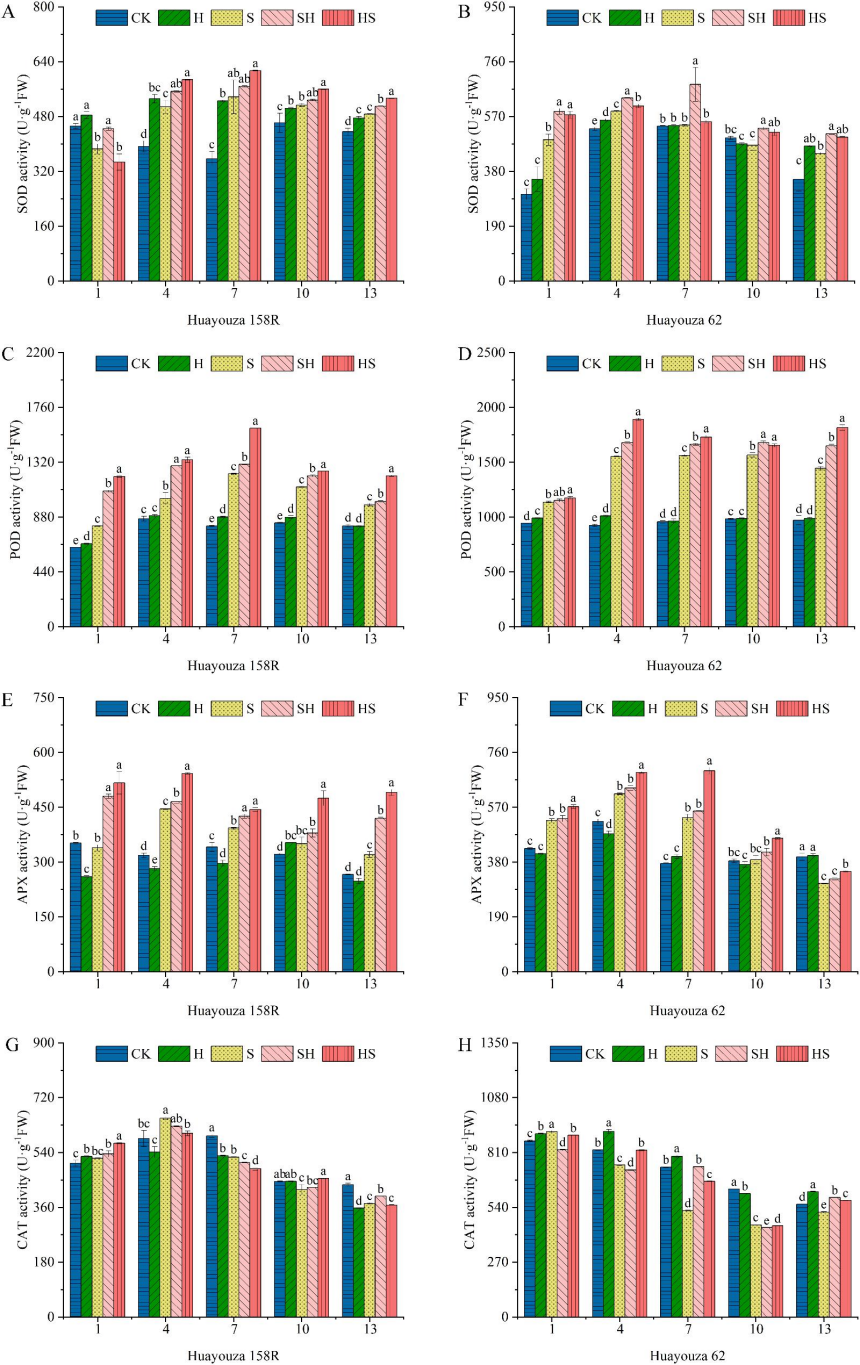
Effect of Hemin prophylaxis and treatment on superoxide dismutase (SOD) (**A**, **B**), peroxidase (POD) (**C**, **D**), ascorbate peroxidase (APX) (**E**, **F**), catalase (CAT) (**G**, **H**) in rapeseed under NaCl stress. CK: Control; H: Hemin; S: NaCl; SH: NaCl + Hemin; HS: Hemin + NaCl. Mean ± SE of three replicates. Different letters indicate significant differences (*p* < 0.05)

Except that the CAT activity in Huayouza 62 decreased with the increase of treatment time, the activities of SOD, POD, CAT and APX enzymes in other treatments increased first and then decreased. Compared with CK, NaCl stress significantly enhanced the activities of antioxidant enzymes (SOD, POD, APX). As the NaCl treatment (S) was applied to plants, there was most significantly enhanced by 50.6% at 7th day and 62.6% at 1st day in SOD, 51.4% at 7th day and 68.1% at 4th day in POD, 39.9% at 4th day and 41.9% at 7th in APX, respectively for Huayouza 158R and Huayouza 62. The SOD, POD, and APX enzyme activities under SH treatment were increased by an average of 7.5%, 16.4%, 15.4% in Huayouza 158R and 7.4%, 18.7%, 4.1% in Huayouza 62 compared with those under S treatment. The HS treatments were enhanced by an average of 12.1%, 9.0%, 28.8% in Huayouza 158R, and 13.4%, 35.1%,16.8% in Huayouza 62 (Fig. 7A-F). In contrast, the CAT activity of Huayouza 158R under NaCl stress was significantly enhanced by 11.4% on the 4th day, and then decreased significantly at the 7th -13th days (Fig. 7G). Hemin treatment significantly enhance CAT activity 1.7% enhancement in SH and 8.7% enhancement in HS at day 10 compared with S treatment. The most significant enhancement was 41.2% and 27.6% in SH and HS treatments, respectively on the 7th day, for Huayouza 62. It means that the activities of antioxidant enzymes in seedlings under different treatments behaved differently. Both Hemin pretreatment (HS) and leaf spraying with Hemin (SH) during stress significantly enhanced antioxidant enzyme activities compared with NaCl treatment alone, and the enhancement was more pronounced for prevention (HS) than for rescue (SH) (except for SOD enzyme activity of Huayouza 62 at the 4th day).

### Effect of foliar spraying of Hemin on the soluble protein of rapeseed seedlings before and after NaCl stress

**Fig. 8:**
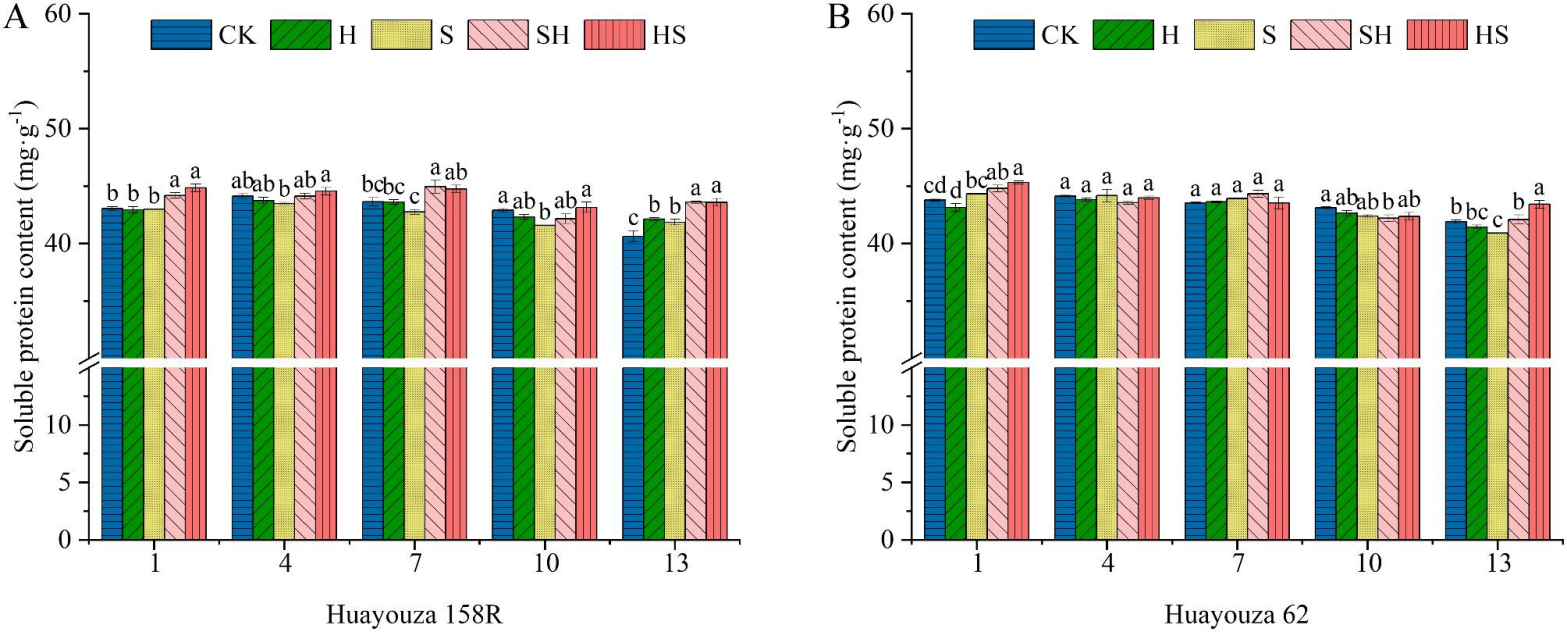
Effect of Hemin prophylaxis and treatment on soluble proteins (**A**, **B**) in rapeseed under NaCl stress. CK: Control; H: Hemin; S: NaCl; SH: NaCl + Hemin; HS: Hemin + NaCl. Mean ± SE of three replicates. Different letters indicate significant differences (*p* < 0.05)

The soluble protein decreased with time under NaCl treatment (Fig. 8). In Huayouza 158R, the soluble protein content was lower than that of the control at days 4–10, and was significantly increased by 3.1%, on the 13th day, under NaCl stress. At days 1 to 13, SH treatments were significantly increased by 1.4-4.2% and HS treatments were significantly increased by 2.5-4.7% compared to NaCl (S) treatment. Huayouza 62 only showed a significant increase of 2.9% in the SH treatment and 6.1% in the HS treatment on the 13th day (Fig. 8B).

## Discussion

Salinity severely inhibits plant morph-physiology and it is important to enhance stress tolerance of plants [23]. Hemin application can mitigate the adverse effects of abiotic stress on plant growth and development [24]. Therefore, we investigated the effects of Hemin foliar application on phenotype, cell membrane, antioxidant enzymes activities, photosynthesis and biomass accumulation of rapeseed seedlings under salinity stress. The results showed that seedling height, root length, stem diameter, and leaf area of rapeseed of both varieties were significantly reduced under salt stress (Table 1; Fig. 1). Exogenous application of Hemin alleviated the inhibitory effect of salt stress. Except for seedling height, which was lower in HS than in SH treatment, the other indexes (root length, stem thickness, leaf area and biomass accumulation) were higher than in SH treatment, and the effect of HS treatment increase was more significant in Huayouza 158R (Table 1; Figs. 2 and 3). It’s indicating that Hemin pre-treatment (HS) achieved the objective of seedling strength. The study suggesting that prophylaxis (HS) is better for alleviating salt stress. Previous studies have shown that Hemin promotes root elongation and lateral root formation [25], consistent with the results of this study. Hemin promotes IAA synthesis [26, 27]. IAA plays an important role in root growth [28, 29].Therefore, it was speculated in this experiment that Hemin might promote root elongation under salt stress by indirectly increasing the accumulation of IAA in the root system.

Photosynthesis plays an essential physiological function in plants’ growth and development, providing various organic substances for their growth, and it is also the most sensitive process of plants after salt stress [30]. Higher salt concentrations increase the accumulation of reactive oxygen species, damage the structure of cysts, stomatal closure, disrupt electron transfer, and inhibit photosynthetic efficiency [31, 32]. Hemin improves photosynthetic efficiency, antioxidant capacity and increases biomass accumulation under heavy metal stress [24]. The present results showed that salt stress significantly reduced stomatal conductance (Gs), net photosynthetic rate (Pn), transpiration rate (Tr), and intercellular carbon dioxide concentration (Ci) in rapeseed. And both HS treatments were higher than the SH treatment (but did not reach significant levels, Fig. 6). Too low stomatal conductance hinders carbon dioxide diffusion (stomatal restriction), inhibits photochemical reactions (non-stomatal restriction), and reduces intercellular carbon dioxide concentration, making the photosynthetic raw material insufficient and reducing photosynthetic rate [33, 34]. The study found that the Gs and Ci values changes in Huayouza 158R were consistent (Fig. 6C, E), indicating that the decrease in net photosynthetic rate may be caused by stomatal limitation [35, 36]. On the contrary, the Gs and Ci values of Huayouza 62 changed oppositely at the 7th-10th day (Fig. 6D, F), indicating that it may be limited by stomata in the early stage and non-stomatal limitation in the later stage, which led to the decrease of Pn. This is consistent with previous studies on the reduction of Pn values by non-stomatal factors under drought and salt stress [37]. Plants under stress form self-protection by reducing water transport [38], and the increase in Tr under Hemin treatment reduces leaf temperature and favors photosynthesis. Therefore, it was concluded that Hemin treatment could improve the photosynthetic capacity by increasing the gas exchange capacity of seedlings under NaCl stress.

High chlorophyll content (SPAD values) increases light absorption to drive the photosynthetic process [39]. Heme oxygenase-1 (HO-1) protects photosensitive pigment clusters and promotes chlorophyll synthesis [40, 41]. As a heme oxygenase-1 (HO-1) inducer, Hemin application can alleviate the inhibition of photosynthesis by heavy metals such as zinc and cadmium [18, 42]. Consistent with the present study, Hemin increased SPAD values, Pn, and accumulation of biomass (Fig. 5, and 6 A, B, and Figs. 2 and 3). In Huayouza 158R, NaCl stress caused a significant decrease in SPAD values. It may be mainly due to two reasons: (i) a decrease in chlorophyll synthesis; and (ii) an increase in chlorophyll degradation [43]. In the Huayouza 62 variety, the SPAD values increased significantly at the 1st-7th days of NaCl treatment, probably due to decreased leaf area and increased leaf thickness [44], which increased relative chlorophyll content per unit of leaf area. In this study, we found the SPAD values about the two varieties of HS treatment was significantly higher than that of SH treatment (Fig. 5). Hemin alone did not increase SPAD values in rapeseed seedlings, probably due to the absence of stress signaling, HO1 was not expressed. In contrast when plants feel salt stress, the activity of HO1 increases, increasing the chlorophyll content of seedlings under stress, thus playing a protective role in photosynthesis. The study demonstrated that Hemin pretreatment was more protective of photosynthesis under salt stress in rapeseed, and was more conducive to plant biomass accumulation and growth and development of rapeseed.

The accumulation of osmoregulatory substances in plant cells is an essential mechanism for plant tolerance to salt stress [45, 46]. The osmoregulation of Hemin focuses on glycan osmoregulatory substances [18, 47]. In this study, the soluble protein content of Huayouza 158R seedlings decreased significantly under salt stress. Hemin increased the soluble protein content. This indicates that Hemin can improve salt tolerance in rapeseed by increasing soluble protein content. Severe salt stress can cause protein degradation or inhibition of protein synthesis [48]. In Huayouza 62 rapeseed, salt stress and exogenous application of Hemin had no significant effect on leaf soluble protein content. This suggests that Hemin may improve the salt tolerance of rapeseed through other osmoregulatory substances.

Plants have evolved a complex antioxidant system in response to abiotic stresses [25]. In this study, NaCl treatment significantly increased the membrane lipid peroxidation level of seedlings and the electrolyte leakage rate of leaves (Fig. 4). The MDA content of both rapeseed varieties reached a maximum on the fourth day and then decreased (Fig. 4C, D). In the early stages of stress, plants may perceive the stress-induced damage and adapt to the stress through an antioxidant defense system or osmotic substance synthesis, thus reducing the MDA content [49, 50]. In our study, salt stress significantly increased SOD, POD, and APX activities in rapeseed seedlings compared with CK and decreased with time (Fig. 7). However, the CAT activity of Huayouza 158R decreased (Fig. 7G, H). It is demonstrated differences in the response pattern of different antioxidant enzymes activities in response to salt stress in plants due to differences in genes, plant species, treatment times and treatments [51, 52]. Previous studies have shown that Hemin activates antioxidant enzymes and reduces MDA content under salt stress [53]. M.A. Shkliarevskyi’s study found that Hemin increased SOD, POD, CAT and APX activity of wheat under heat stress [19, 54]. n our study, Hemin reduced membrane damage, increased SOD, POD, and APX activities. Especially the POD and APX activities were significantly higher in HS treatment than in SH treatment in both varieties, while the opposite was true for SOD activity in Huayouza 62 (Fig. 7B-F). This indicates that the regulatory mechanism of Hemin differs in medium salt tolerance Huayouza 158R and strong salt tolerant Huayouza 62 varieties. The current studies have found that Hemin can alleviate the oxidative damage by enhancing the antioxidant capacity [17], promoting HO1 expression, and its metabolite Fe^2+^, CO enhances antioxidant enzyme activity in the plant, which participates in the ROS scavenging process and mitigates the damage to the plant [55]. Consistent results were shown under zinc stress in rice, salt stress, and cadmium stress in cabbage [17, 18]. However, whether Hemin plays a role in rapeseed salt tolerance through its metabolites and the regulatory mechanism of prevention and treatment remains to be further elucidated.

## Conclusions

In conclusion, we have demonstrated that under salt stress, exogenous Hemin treatments can drastically increase antioxidant enzymes activities such as SOD, POD and APX, effectively reduces membrane oxidative damage. Furthermore, exogenous Hemin enhanced morph-physiological traits such as photosynthesis in rapeseed leaves, delayed leaf senescence, and improved dry matter accumulation and distribution under salt stress. The results of this study showed that the effect of Hemin pretreatment (HS) to alleviate salt stress in rapeseed was more significant. Thus, Hemin can be used as effective inducer to enhance the tolerance of rapeseed seedlings to salt stress and improve the sustainability of rapeseed production in saline soils. In agricultural applications, preventive measures can be adopted to enhance rapeseed salt tolerance, and increase crop economic benefits.

## Materials and methods

### Design of the experiment

The *Brassica napus* (AACC, 2n = 38) moderately salt-tolerant variety Huayouza 158R and strongly salt-tolerant variety Huayouza 62 were selected in this investigation. The seeds provided by Academician Fu Tingdong’s team from Huazhong Agricultural University. The plant growth regulator Hemin (provided by Shanghai Changdeduo Agricultural Technology Co., Ltd.) was used for foliar application. The pot experiment used completely randomized block design was conducted in 2022–2023 at the daylight linkage greenhouse (under natural light, 25/20 ± 2 °C day/night temperatures, 60% relative humidity) of Binhai College of Agriculture, Guangdong Ocean University (N: 21°8′56 ″, E: 110°17′58″, ASL: 20 m).

Fully mature and uniform seeds were chosen manually and sterilize with 3% hydrogen peroxide for 10 min, then thoroughly rinse 3–5 times with distilled water. Seeds were sown in plastic seedling trays (54 cm × 28 cm) containing 32-hole (4 × 8), each per hole (with an upper diameter of 6 cm, lower diameter of 2 cm, height of 11 cm, and no holes in the bottom) filled with about 0.12 kg of test soil (a mixture of vermicompost and sand (3:1, v:v)). 2 seeds were sown in each hole. Interplanted at one true leaf and retained 1 seedling per hole. Water 1/2 Hoagland nutrient solution once at the growth of two true leaves, 20 ml per hole, and no more nutrient solution at a later stage.

The treatments were carried out at three true leaves, and morphologically similar plants were selected and divided into five groups: (1) CK (clear water, foliar spraying with distilled water); (2) H (clear water, foliar spraying with 5 µmol/l of Hemin); (3) S (0.6% NaCl sodium, m salt: m soil = 6:1000 brine, distilled water); (4) SH (NaCl sodium; foliar spraying with 5 µmol/l of Hemin after NaCl stress 24 h); (5) HS (foliar spraying of 5 µmol/l of Hemin before NaCl stress 24 h; NaCl sodium ), where the salt treatment was watered by dissolving a quantitative amount of NaCl into 20 ml of water per hole. Treatments were carried out at 7:00 p.m., where salt and Hemin were co-treated at 24 h intervals, and all salt treatments and Hemin alone were carried out on the same day, and all treatments were completed in 3d. Sampling was done on the 1st, 4th, 7th, 10th, and 13th days after completion of all treatments, 3 replicates per treatment.

### Measurement items and methodology

#### Measurement of growth parameters

Rapeseed seedling height and root length were measured with a straightedge. Stem thickness at the cotyledon scar was measured with vernier calipers, and fresh weights of above-ground and below-ground parts were weighed with an electronic balance. Fresh samples were killed in an oven at 105 °C for 30 min, dried at 75 °C to a constant weight, and weighed to determine the dry weights of above-ground and below-ground parts.

#### Determination of electrolyte leakage rate

Electrolyte leakage (EL) was determined according to the method described by Dionisio-Sese and Tobita [56]. For each treatment of 5 plants, leaf tissues from the same site were selected and cut to 5 mm size, weighed 0.1 g placed in test tubes containing 10ml of deionized water, and left at room temperature (25 °C) for 24 h, the conductivity of the solution was measured using a conductivity meter (E1), the samples were heated for 30 min in a thermostatic water bath at 100 °C, cooled down and then the conductivity of the solution was measured again (E2). The EL value was calculated using the following The EL value is calculated using the following formula:

$${\rm{Electrolyte}}\,{\rm{Leakage}}\,{\rm{ = }}\,\left( {{\rm{E1/E2}}} \right)\,{\rm{ \times }}\,{\rm{100\% }}$$

#### Analysis of lipid peroxidation and membrane permeability in leaf blades

The concentration of malondialdehyde (MDA) was measured according to Stewart and Bewley [57]. Fresh leaf samples were separated from the growing branches and ground with a mortar in a mortar containing 5 mL of TBA 0.6% and 10% TCA. The mixture was heated at 100 °C for 15 min. In the next step, the samples were cooled on ice for 5 min and centrifuged at 5000 rpm for 10 min, and the absorbance of the supernatant was measured at 450 nm, 532 nm, and 600 nm.

MDA content was calculated based on fresh weight as follows:

$${\rm{(NMOL}}\,{\rm{MDA}}\,{\rm{ g^{-1}}}\,{\rm{FW)}}\,{\rm{ = }}\,{\rm{6}}{\rm{.45}}\,({\rm{OD532}}\,{\rm{ - }}\,{\rm{OD600}})\,{\rm{ - }}\,{\rm{0}}{\rm{.56}}\,({\rm{OD450}})$$

#### SPAD and gas exchange parameters

SPAD values were determined on the 1st, 4th, 7th, 10th, and 13th days using the American photosynq MultispeQ multifunctional phytometer. Net photosynthetic rate (Pn), stomatal conductance (Gs), intercellular CO_2_ concentration (Ci), and transpiration rate (Tr) of the functional leaves were measured daily from 9:00 to 11:30 a.m. at the end of all treatments on the 1st, 4th, 7th, and 10th days using a Li-6400 portable photosynthesize (LI-COR, Inc., USA). Leaf chamber conditions were: photosynthetically active radiation of 1000 µmol m^− 2^ s^− 1^, flow rate of 500ml/s, and relative humidity of 60-70%.

#### Determination of antioxidant enzyme activity

The preserved fresh leaves (0.5 g) were ground with liquid nitrogen and 10 ml of pre-cooled phosphate buffer (50 Mm; PH 7.8) was added in two batches, ground to a homogenate and poured into a centrifuge tube and centrifuged for 20 min at 4 °C and 10,000 g. The supernatant was taken to determine the activities of catalase (CAT), superoxide dismutase (SOD), peroxidase (POD), and ascorbic acid peroxidase (APX). SOD activity was calculated by measuring its ability to inhibit nitrotetrazolium blue (NBT) photoreduction as described by Giannopolities and Ries [58]. Referring to the method of Stewart and Bewley [57], 0.1 ml of enzyme solution was mixed with 2.9 ml of reaction solution and subjected to light reaction at 4000 lx for 20 min at 25 °C and then absorbance at 560 nm was measured using unilluminated tubes as a control. For POD activity, refer to the method described by Klapheck, Zimmer, and Cosse [59]. The absorbance values of the reaction mixture at 470 nm were determined by the guaiacol method as described and counted every 30s. CAT activity was calculated by measuring the decomposition of H_2_O_2_ at 240 nm according to Gupta et al. [60], Li et al. [61]. APX activity was calculated according to Nakano and Asada [62].

#### Osmoregulatory substances

The soluble protein content was measured according to the method of Bradford [63], and the absorbance value at 595 nm was determined by binding the proteins with Caumas Brilliant Blue.

### Statistical analysis

Excel 2016 was used for data organization and collection, one-way ANOVA and Duncan’s test (p < 0.05) were used to analyze the data using IBM SPSS Statistics 26 software, and origin 2021 software was used for plotting.

## Data Availability

The datasets used and/or analysed during the current study are available from the corresponding author on reasonable request.
